# Voronoi Tessellation Captures Very Early Clustering of Single Primary Cells as Induced by Interactions in Nascent Biofilms

**DOI:** 10.1371/journal.pone.0026368

**Published:** 2011-10-18

**Authors:** Iris Hödl, Josef Hödl, Anders Wörman, Gabriel Singer, Katharina Besemer, Tom J. Battin

**Affiliations:** 1 Department of Limnology, University of Vienna, Vienna, Austria; 2 WasserCluster Lunz GmbH, Lunz am See, Austria; 3 Baden, Austria; 4 Department of Land and Water Resources Engineering, Royal Institute of Technology, Stockholm, Sweden; Netherlands Institute of Ecology, Netherlands

## Abstract

Biofilms dominate microbial life in numerous aquatic ecosystems, and in engineered and medical systems, as well. The formation of biofilms is initiated by single primary cells colonizing surfaces from the bulk liquid. The next steps from primary cells towards the first cell clusters as the initial step of biofilm formation remain relatively poorly studied. Clonal growth and random migration of primary cells are traditionally considered as the dominant processes leading to organized microcolonies in laboratory grown monocultures. Using Voronoi tessellation, we show that the spatial distribution of primary cells colonizing initially sterile surfaces from natural streamwater community deviates from uniform randomness already during the very early colonisation. The deviation from uniform randomness increased with colonisation — despite the absence of cell reproduction — and was even more pronounced when the flow of water above biofilms was multidirectional and shear stress elevated. We propose a simple mechanistic model that captures interactions, such as cell-to-cell signalling or chemical surface conditioning, to simulate the observed distribution patterns. Model predictions match empirical observations reasonably well, highlighting the role of biotic interactions even already during very early biofilm formation despite few and distant cells. The transition from single primary cells to clustering accelerated by biotic interactions rather than by reproduction may be particularly advantageous in harsh environments — the rule rather than the exception outside the laboratory.

## Introduction

Spatial configuration is fundamental in defining the structural and functional properties of biological systems [1], [2]. Biofilms, surface-attached and matrix-enclosed microbial communities [3]–[5], are a striking example of spatial organisation. Multicellularity and collective behaviours [6], [7], such as genetic [4], [8] and physiological [9] specialization are increasingly recognized as the pillars of the success of the biofilm mode of life. Conceptual models [3], [9], [10] have stimulated substantial work aiming at elucidating biofilm formation, but the early steps from early adhesion of single primary cells towards an organized life remain poorly understood. On the one hand, research on early biofilm formation mostly focuses on the physicochemical mechanisms [11], [12] and genetic regulation [13] of cell adhesion. On the other hand, studies on multicellularity and microscale spatial structures of surface-attached microorganisms remain restricted to dense and reproducing monocultures in nanofabricated landscapes [14]–[19] or extracellular scaffolds [20]. Today, the transition from single primary cells towards multicellularity in natural biofilms remains elusive.

Biofilm theory [21] and biofilm modelling [22], [23] generally assume randomly-seeded surfaces as initial conditions for biofilm formation, an assumption that is analogous to the deposition of colloidal particles and droplets onto a homogeneous surface [24]. In the absence of reproduction and interactions, initial uniform randomness persists even in a finite space [21] given the random walk of bacteria in a homogeneous fluid [25], [26]. Only if cells start to reproduce and to die [27], or to interact once a certain abundance has been reached [28], their spatial distribution may deviate from randomness. It is therefore reasonable to interpret the gradual deviation from uniform randomness as a signature of the transition from single-cell life towards cell clusters, which form the basis for microcolonies and for even more complex biofilm entities.

We employed Voronoi tessellation (VT), widely used in crystallography, geography and astrophysics [29]–[31], to quantify the spatial distribution patterns of cells and non-living particles (NLP) in an experimental stream. NLPs, a complex mix of organics and minerals, are abundant in streams, and constitute important building blocks of stream biofilms [32]. VT is a geometrical method partitioning space based on ‘seeds’ (i.e., points with explicit spatial position) by assigning any point in space between and around seeds to the closest seed. This simple geometrical rule generates polygons differing in shape and size, both of which depend on distances among all neighbouring seeds and thus represent an intuitively reasonable partitioning of the space over which seeds may interact [33]. Under the assumption of no interactions among seeds, the population of polygon sizes generated by VT follows a Poisson-Voronoi (PV) uniform random distribution [34]. In this study, we chose Voronoi tessellation rather than “nearest neighbour” methods (27), as Voronoi tessellation allows the determination of the natural neighbours for each individual seed and of the distance among seeds. Our results show that cell and NLP distribution on initially sterile glass slides deviated from a uniform random distribution almost immediately after the exposure of the slides to the natural microbial community in the stream water. Depending on the exposure of cells to different hydrodynamic microenvironments, the gradual deviation from uniform randomness became even more pronounced as colonization progressed. A mechanistic model that assumes interactions, such as cell-cell signalling or chemical surface conditioning by manoeuvring cells, successfully predicted the observed deviation from the Poisson-Voronoi distribution.

## Materials and Methods

### Hydrodynamic environment

**Figure 1:**
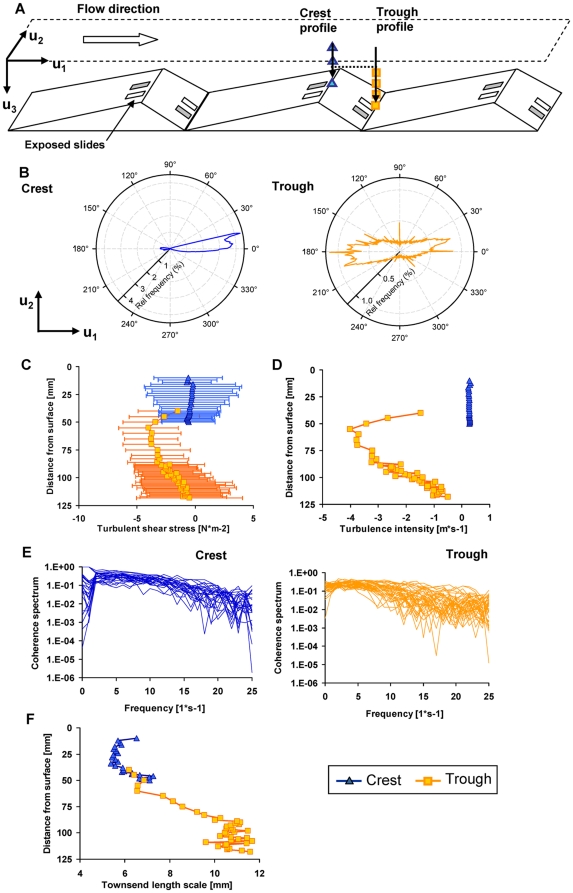
Characterisation of the hydrodynamic microenvironment at the crest and in the trough. (**A**) Location of the glass slides used for microbial colonization at the crest and in the trough of bedforms. (**B**) Polar plots showing the frequencies of two Cartesian components (downstream = *u*_1_, lateral = *u*_2_) of the flow velocity revealed that flow was predominantly directed downstream at the crest but was multidirectional in the trough. From each depth, turbulence intensity, shear stress, turbulence structure and microscopic length scale of turbulent eddies were computed. (**C**) Throughout the water column, shear stress was higher and more variable in the trough than at the crest. (**D**) Turbulence intensity was generally higher in the trough than at the crest. (**E**) Coherence spectra describing the relationship between the time series of the longitudinal and vertical flow dominating the longitudinal shear stress showed more structured flow at the crest than in the trough. (**F**) Microscopic length scale of turbulent eddies.

A flow landscape typical for streams with low submergence was mimicked in a streamside flume (L: 40 m, W: 0.40 m). Graded and periodically installed bedforms (bottom length: 1 m; width: 0.40 m; ascending slope: 0.75 m; descending slope: 0.25 m; maximum elevation: 0.08 m) induced variable flow over the whole flume length. The flume was fed in a once-through mode with stream water (Oberer Seebach, Austria) containing the natural microbial inoculum (not characterized). The flow rate was adjusted to 2.25 L s^−1^ with an average flume-scale flow velocity of 0.08 m s^−1^; average residence time of water was 8 minutes in the flume. The crest and the trough were selected as hydrodynamic extremes (**Figure 1a**). High-resolution Acoustic Doppler Velocimetry (ADV, Nortek Vectrino, side-looking probe) was used to capture the 3-dimensional flow velocity over the bedforms. Turbulence intensity, shear stress, turbulence structure and microscopic length scale of turbulent eddies were computed.

The velocity vector (*u*_1_,*u*_2_,*u*_3_) (m s^−1^) was registered in terms of the three Cartesian components (where *u*_1_ is along flume length, *u*_2_ is along flume width *u*_3_ is along flume depth) with a frequency of 50 Hz (time series: 76 s, yielding 4180 single data points per vector). For each velocity component, the mean value was calculated as 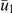 = E[*u*_1_], where E[…] denotes the arithmetic average operation over the time series. A vertical profile (*u*_3_) was measured in increments of 1 mm from 5 mm to 15 mm above the substratum at both locations, crest and trough, respectively. The next 30 mm of water column were measured in 2 mm intervals and only for the trough the last 35 mm where measured in an interval of 5 mm. Turbulence intensity was computed as the root-mean-squared deviation from the mean, i.e. {E[(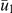
−*u*_1_)^2^]}^0.5^ (m s^−1^), which is the standard deviation of the velocity component; (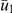
−u_1_) is the velocity fluctuation, also denoted *u*′ (m s^−1^).

Turbulent shear stress, τ (causing eddy viscosity), was obtained as τ_12_ = ρ E[*u*′_1_ *u*′_2_] (N m^2^) for each of the components τ_12_, τ_23_ and τ_13_, where E[*u*′_1_ *u*′_2_] is the covariance between the velocity components (*u*_1_,*u*_2_,*u*_3_). By separating the covariance on harmonic frequencies using the Welsh's method [35], we express the cross-spectrum of the velocity fluctuations *u*′_1_ *u*′_2_, *u*′_2_ *u*′_3_ and *u*′_1_ *u*′_3_. This informs on the distribution of turbulent energy on the frequency of eddies.

A form of microscopic length scale of turbulent eddies was estimated from Townsend length scale [36] according to
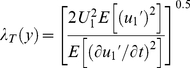
(1)where *U*_1_ is the mean velocity both over time and depth. Since the flow is non-uniform, due to the bedforms, the selection of this scale velocity affects the possibility to compare the relative magnitude of the length scales from each of the two verticals measured above the crest and the trough (**Figure 1a**). Therefore, we have used both a mean velocity for each vertical profile, but also the same mean flow velocity (discharge divided by free cross-sectional area at the crest).

### Colonisation, microscopy and imaging

Replicated initially sterile glass slides that served as substratum for microbial colonisation were exposed near the crest and in the trough of consecutive bedforms within the lower reach of the flume. We used both uncoated slides and also slides coated with polylysine (PLL) as substratum. PLL enhances cell adhesion without stimulating biological activity [37] and were therefore used PLL-slides to test for possible effects of the adhesion environment. Sub-sets of uncoated slides (25 mm×75 mm) were retrieved after 0.5, 1, 2, 3, 4, 5, 6, 8, 10 and 12 h. We selected this narrow time window to preclude cell reproduction from our analyses. The absence of cell reproduction was confirmed by the absence of dividing cells and the absence of the formation of microcolonies as inspected by microscopy (600× magnification). The 12-h-colonisation period is also below the typical doubling time for cells reported from cold-water streams [38], [39] such as ours. Immediately after retrieval from the flumes, microbial cells on the slides were stained with the nucleic acid stain 4′,6-diamidin-2′-phenylindol-dihydrochlorid (DAPI, Invitrogen) and cells visualized with epifluorescence microscopy (Zeiss AxioImager; 100× magnification); dark field microscopy served to visualize NLP. TIF-images (2584×1936 pixel, where one pixel corresponds to 0.31×0.31 µm) of cells and NLP were analysed using public domain software ImageJ (http://rsb.info.nih.gov/ij/index.html); numbers of images and seeds are given in **Table S1**. Color images were split into the red, green and blue channels, thus resulting in 3 individual images with 256 grey values each. Converting grey scale images to binary images, the upper and lower limit for the threshold was adjusted manually on 15 representative images for each individual sample. To differentiate cells and NLP from the background, a mean threshold with grey-value from 31–255 for cells in the green channel and for NLP in the red channel was derived and applied to all images. For cells and NLP, the centre of mass was calculated and used as seed coordinates for Voronoi tessellation.

### Voronoi tesselation

When VT is applied to uniform randomly distributed seeds, a probability density function (PDF) of the resulting polygons can be empirically constructed and used as a reference curve. This Poisson-Voronoi (PV) reference curve is given by 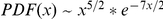, where x represents polygon size [34]. In a first step, we tested if the PV function, as suggested by reference [34], can indeed be transferred to discrete digital images as used in this study. To generate the VT curves as the PDFs of Voronoi polygon sizes, we used a topologically discrete set of elements in a defined 2D Euclidean space (i.e., a digital image with smallest unit one pixel). We mirrored the image at its borders to correct for its finite nature. Simulations of mirrored images with low seed densities (<100 seeds) but accordingly high number of images (5×10^8^ polygons) revealed no significant difference (Kolmogorov-Smirnov test, P<0.001) compared to the empirically derived PV curve [34]. Measured Voronoi areas were scaled so that the average polygon size in the PDF adjusts for differing seed number between images of one treatment and preclude crowding effects due to increasing number of seed in time.

From the PV reference curve we assumed, similar to reference [34], that the PDF of Voronoi polygon sizes for our images is a continuous and smooth function. Therefore, instead of binning the measured and scaled Voronoi areas, we constructed a cumulative probability function (CPF) (**Figure S1**) without loss of information. The endpoint of each segment was given by
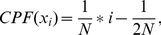
(2)where *N* is the total number of measured Voronoi areas, *x_i_* is the i^th^ measured and scaled Voronoi area sorted by size. The values of CPF(x) for all x in the intermediate segments were linearly interpolated between the endpoints x_i_ (electronic supplementary material Figure S1). The cumulative function of the Poisson-Voronoi reference curve CPV(x) is proportional to the lower incomplete gamma function (γ) for 7/2; CPV(x)∼γ_7/2_(x*7/2). Therefore, we assumed the initial (i.e., very small Voronoi polygons) segment of the CPF to be proportional to the appropriate lower incomplete gamma function CPF(x)∼γ_7/2_(x*7/2) for all x<x_1_ and the values of CPF(x) in the final ( i.e., very large Voronoi polygons) segment to be proportional to the upper incomplete gamma function (Γ) (1−CPF(x))∼Γ_7/2_(x*7/2) for all x>x_N_. The proportionality factors are determined so that there is no discontinuity in the CPF. This CPF is a simple continuous description of the empirical data. We calculated individual CPFs for all images, which were then pooled and averaged for each flow environment (i.e., crest versus trough) and colonization phase (i. e., 0.5 h to 12 h). The PDF was then calculated by numerical differentiation of the CPF. We defined the derivative in a chosen interval 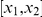 (**Figure S1**) as
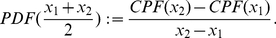
(3)The values were read in an interval of 0.01 resulting in a total of 700 function values for the PDF. Kolmogorov-Smirnov statistics served to test for differences among the observed and the PV reference distribution functions.

### Modelling VT curves

**Figure 2:**
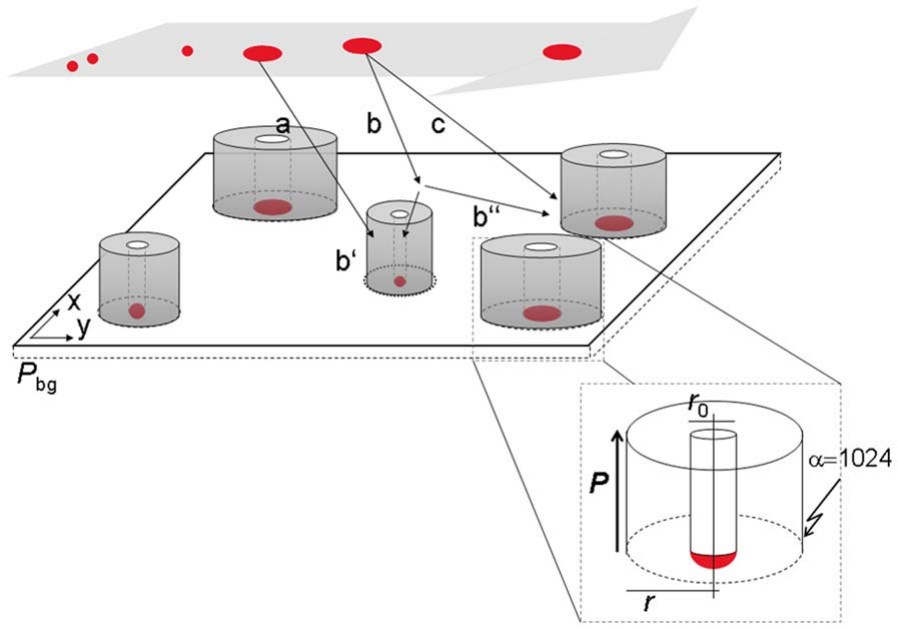
Heuristic model depicting a landscape with seeds and their interaction strength as used for modelling the Voronoi tesselation curves. The background attraction (*P*_bg_) of a given surface area (*XY*) increases as seeds with a given interaction strength cluster onto that surface. Thereby, they increase the probability of new seeds to colonize within their vicinity. The interaction strength of a given seed is essentially given by *r*_h_, the inner radius, *r*_0_, and the slope, α. To simplify the model we set α = 1024 resulting in a cylindrical volume. A given seed, still suspended in the water, can be directly attracted to an attached cell (path a) or posterior to its initial adhesion (b) by a neighbouring cell (paths b′ and b″), or it can attach without any interaction (c) but will provide attraction for the following seeds.

In the following, we describe the mechanistic model we used to simulate the observed VT curves. Essentially, we assume that interactions among seeds generated, at least in part, the observed spatial patterns of cells and NLP. As illustrated in **Figure 2**, the model envisages in a first step that an initial seed attaches randomly onto the surface. This attachment influences the probability of subsequent seeds to attach in the vicinity of the initial seed. This will ultimately lead to clustering irrespective of the nature of the interactions. The model thus assumes increased probability of a cell or NLP to adhere in the vicinity of a seed compared to the background. We treat the background probability landscape initially as a uniform flat slab and the interaction strength of each newly attached seed changes this probability landscape. The increase of additional relative probability is given by
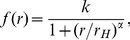
(4)where *r* is the radius within which the attached seed affects the probability, *k*, of a next seed to attach, *r_H_* is *r* at *k*/2 and thus describes the size of the area the attached seed may interact with a not yet attached primary cell (or NLP), and the slope α describes the probability distribution across *r* (**Figure 2** and **Figure S2**). By rotating *f(r)* around the vertical axis, we obtain the attachment probability over the surface area as defined by *r*. The resulting “volume”, *V*, translates into the interaction strength of a given seed, and *k* can be derived from
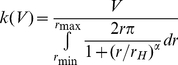
(5)We assumed the probability for a cell or NLP to attach on top of a seed to be zero. Therefore, the closest proximity for attachment of a new primary cell next to a seed is expressed by the inner radius, *r*_min_, and *f*(*r*) equals zero for *r*<*r*_min_. A slope, α = 0, has no boundary and extends to infinity but α>10 starts to approximate the shape of a cylinder (**Figure S2**). Setting α = 1024 resulted in a cylindrical attraction volume where *r* = *r*_H_ = r*_max_* (**Figure S2**) and with a statistically homogeneous attraction probability, *k*, along *r*. Based on this cylindrical attraction volume, spatial patterns of seed distribution were simulated and validated.

We did not attempt to identify the actual nature of the interactions among seeds. However, for cell-to-cell signalling, for instance, we would expect α to approximate a decay function (0<α<10; **Figure S2**) that describes signal propagation away from the emitting seed [40]. Manoeuvring cells may leave an extracellular polymeric footprint that conditions the surface area proximate to the cell itself [41], [42]. We assume that, averaged over a large number of primary cells, this footprint would result in a cylindrical attraction volume with constant α. We considered this as a parsimonious approach integrating over the diverse behaviours (e.g., motility, signalling, EPS production) in a complex microbial community.

To simulate the spatial patterns of seed distribution and to compare these patterns with the measured VT curves, we distributed the actual number of seeds of each treatment and sampling date with identical interaction strengths over a discrete surface identical to the size of the images (*XY*-axes, 5×10^6^ pixels) to create a probability landscape (**Figure 2** **; Table S1**). The background probability (*P*_bg_) landscape without seeds is uniform and flat, and has a total probability volume of 500×10^6^ as we uniformly assigned a *P*_bg_ of 100 to each pixel. A first seed with a given interaction strength is randomly placed onto the uniform landscape, changes the probability topography and therefore attracts the subsequently deployed seeds. Once all seeds are deployed in the landscape, they increase the overall landscape attraction probability by number of seeds times the interaction strength. This image generating procedure was repeated for each image simulating a total of 16×10^4^ seeds to ensure an accuracy of 0.0025 for each simulation. The resulting images were processed in the same way as the microscopic images to produce a modelled VT curve. Finally, a residual sum of squares computed over the 700 differentiation intervals of the modelled and empirical VT curves was computed as a fitting criterion to be minimized over multiple model runs with different parameter sets. Extensive modelling with interaction strengths *V* ranging from 0.25×10^6^ to 20×10^6^ per seed and the *r_H_* varying from 5–80 µm was performed and optimum fits identified and checked by contour plots of residual sums of squares as a function of *V* and *r_H_* (**Figure S3**).

## Results

### Hydrodynamic environment

We begin the results by describing the hydrodynamic microenvironment at the crest and in the trough of consecutive bedforms (**Figure 1a**). In fact, it is becoming increasingly recognized that even individual microbial cells can sense small-scale changes in turbulence [12], [43], [44] and that the spatial variation of small-scale hydrodynamics can affect stream biofilms [45].

Flow was predominantly unidirectional at the crest and multidirectional in the trough (**Figure 1b**). Longitudinal turbulent shear stress was consistently lower throughout the entire water column above the crest (0.63±0.06 N m^−2^) than in the trough (0.81±0.19 N m^−2^) (**Figure 1c**) for a region between 5 and 10 mm above the bottom. At the crest, the laminar shear stress was estimated from the vertical velocity gradient at 0.001 N m^−2^ and was thus markedly lower than the turbulent shear stress. This suggests that turbulent mixing dominated over molecular mixing close to the bottom where glass slides were exposed for primary colonization. The average temporal variation of the turbulent shear stress was 5 times higher in the trough than at the crest. Correspondingly, turbulence intensity was generally higher in the trough than at the crest (**Figure 1d**). The steeper slope of the coherence spectra and their lower variation through the water column at the crest indicate a more structured turbulence at the crest; the distribution of eddy sizes was more random in the trough (**Figure 1e**). Finally, Townsend microscopic length-scales (**Figure 1f**) suggest larger eddy size in the trough than at the crest.

### Spatial distribution of cells and NLP

**Figure 3:**
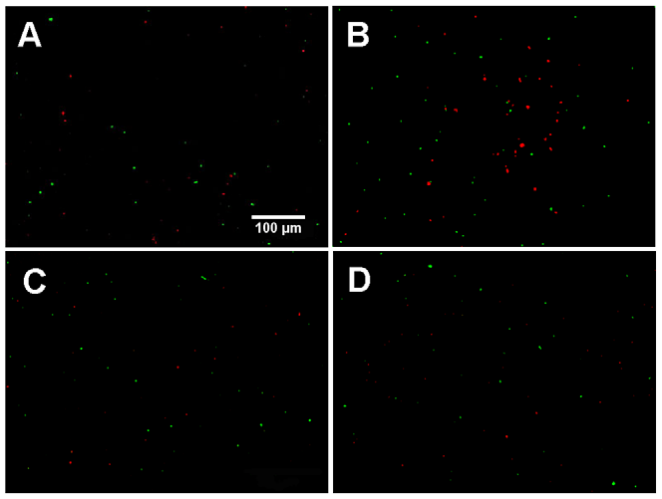
False-color images of cells and NLP. Cells (green, DAPI-stained) and NLP (red) after 0.5 h and 12 h colonisation at the crest (**A, B**) and in the trough (**C, D**), respectively. Clustering is not detectable upon simple visual inspection of these low-abundance and distant seeds.

**Figure 4:**
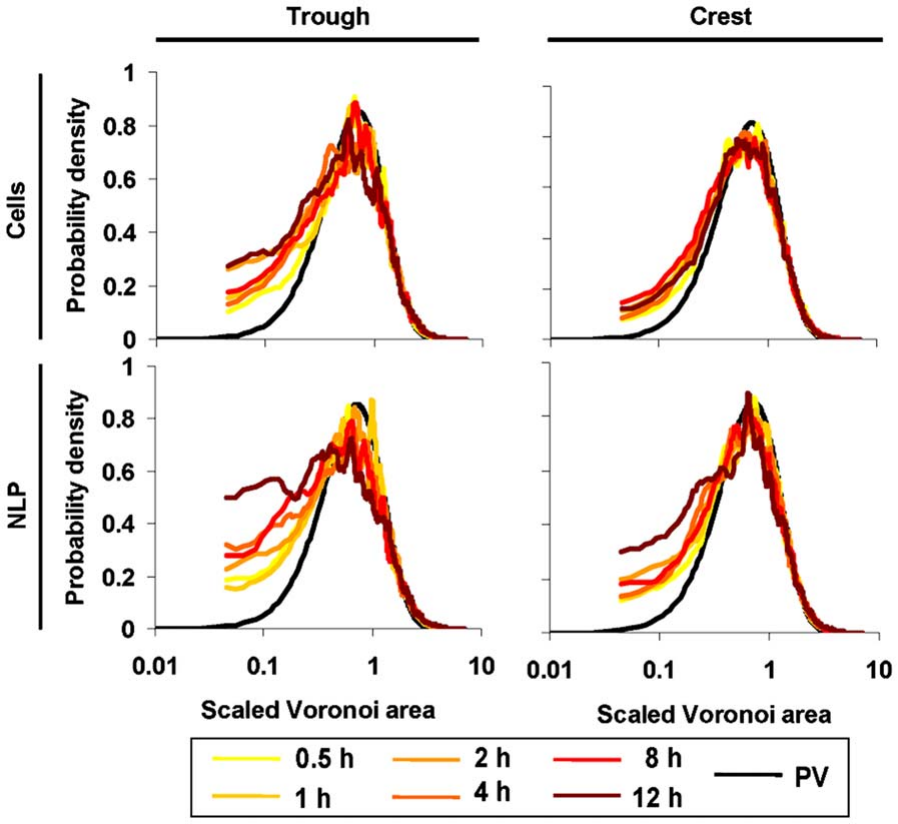
Examples of observed Voronoi tessellation (VT) curves. The distribution pattern of both cells and NLP deviate from the reference curve [34] in the trough and at the crest.

Abundance of cells (37.1±23.0 to 84.1±39.3 cells mm^−2^) and of NLP (18.2±7.5 to 62.0±69.9 NLP mm^−2^) remained generally low at the crest and in the trough, which we attribute to a certain turnover of cells and NLP (see also **Figure S4**). As shown in **Figure 3**, clustering of cells and NLP was not obvious from simple visual inspection of the images. However, VT curves consistently revealed that cells and NLP did not follow a uniform-random PV distribution — even not after 0.5 h colonisation time. Deviation was most prominent for smaller polygons, where both cells and NLP increasingly and consistently deviated from the uniform-random Poisson-Voronoi distribution over the 12-h colonization experiment (**Figure 4**). At the lower end of the VT curves, probability densities for cells were more variable than for NLP over the 12-h colonization period.

### Modelling VT curves

**Figure 5:**
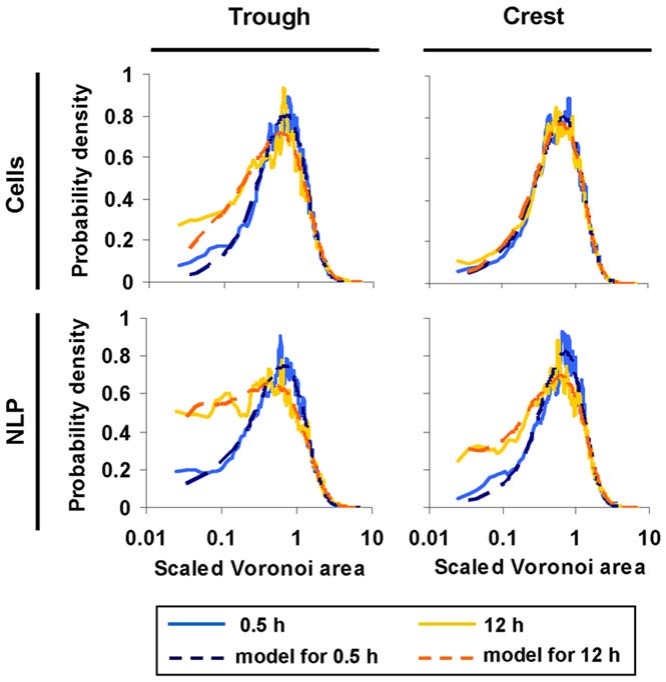
Comparison of observed and modelled VT curves at 0.5 h and 12 h. The respective Kolmogorov-Smirnov statistics of the fits are given in Table S1 for cells and NLP on the crest and in the trough.

**Figure 6:**
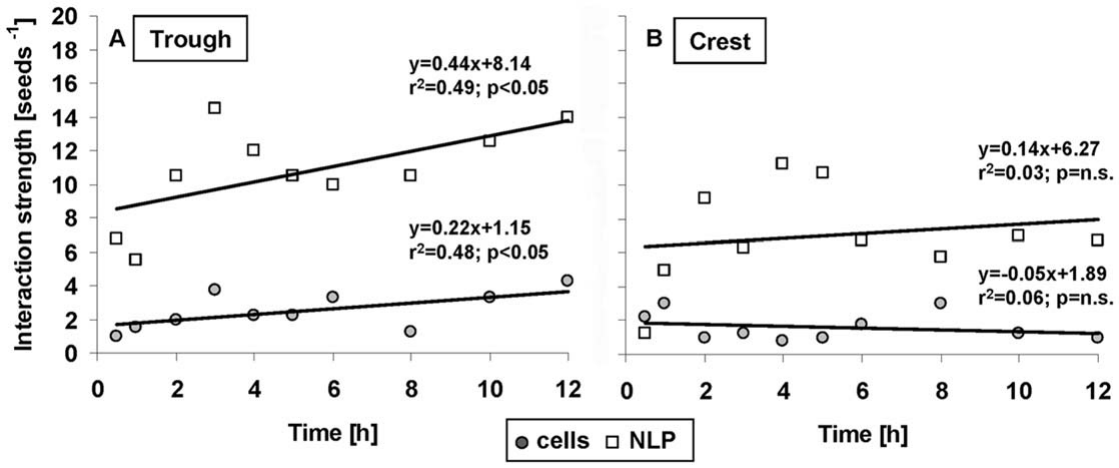
Seeds increasingly deviate from uniform random distribution with colonisation. Relationships between the dimensionless interaction strength per seed and colonization time of cells and NLP on non-coated slides in the trough (**A**) and at the crest (**B**). Given are least squares regression models.

The model predicted the VT curves of the cells and NLP reasonably well, and hence also their deviation from uniform randomness (**Figure 5** **; Table S1**). Fits were particularly good for the interaction strength of cells and NLP, while they were poor for cell *r*_H_ but good for NLP *r*_H_ (**Figure S3**). We suggest that this is attributable to a more variable behaviour of cells (e.g., motility, EPS production) than of NPL. If the deviations from uniform-random Poisson-Voronoi distribution were caused by interactions, then we would expect a gradual deviation from randomness over the 12-h colonization. We found this expectation supported in the trough but not in the crest (**Figure 6**). Simulated interaction strengths of both cells and NLP significantly increased over time in the trough (**Figure 6a**), but not at the crest (**Figure 6b**). The interaction strength was consistently higher for NLP than for cells in both the trough and the crest. PLL-slides generally attracted more cells than uncoated slides (**Figure S4**). In the trough, cell interaction strength increased similarly for both uncoated slides and PLL-slides (comparison of slopes by ANCOVA interaction term: F_1,12_ = 1.8, P = 0.20). At the crest, the interaction strength of cells on PLL-slides significantly (r^2^ = 0.98, slope = 0.32, P<0.001) increased over the 12-h colonization period and thus differed from the uncoated slides (ANCOVA interaction: F_1,12_ = 9.3, P<0.05). The interaction strength of NLP on PLL-slides did not increase significantly over the colonisation period neither in the trough nor on the crest.

### Distance among neighbouring seeds

**Figure 7:**
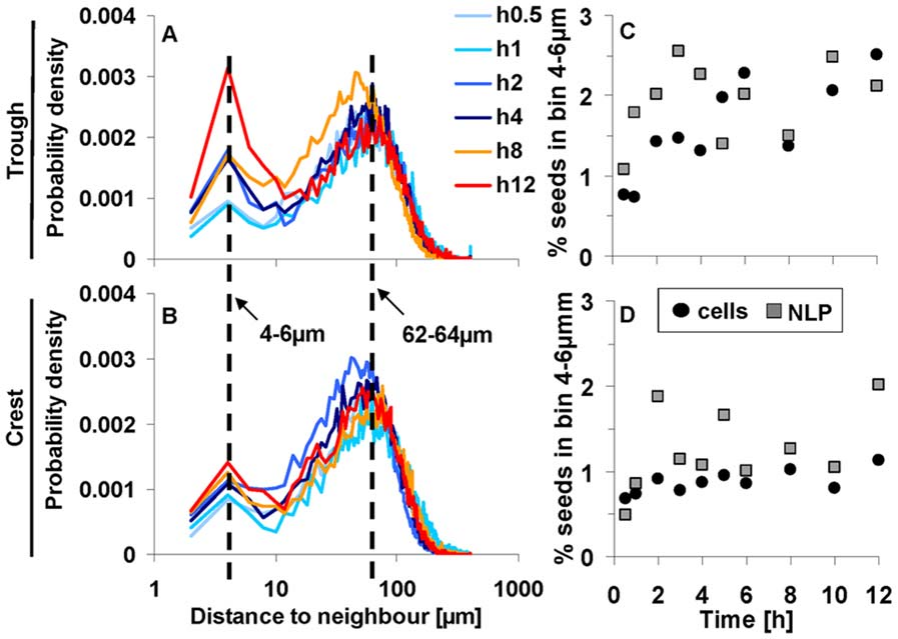
Representative bimodal distributions of neighbour distance of cells in the trough (A) and at the crest (B). The highest values of the secondary peak were, for all seeds in the trough and at the crest, within the 4 to 6 µm bin; the primary peak was more variable around 60 µm. The relative numbers of distances within the 4 to 6 µm peak increased over time in the trough (**C**) but less at the crest (**D**).

VT allows the identification of the neighbours of a given seed [30], [46], [47]. We found on average 5.04±1.54 and 4.49±1.51 neighbours for cells and NLP, respectively; no difference was found between crest and trough. By identifying the respective neighbours, we were able to compute the physical distances among cells (**Figure 7a,b**). Most distances were contained within a secondary peak around the 4-to-6-µm bin and within a prominent peak around the 62-to-64-µm bin (**Figure 7**). The position of the primary peak (62 µm) depends on seed density, and the position of the secondary peak at (5 µm) is typical for seed aggregation. Probability densities for the secondary peak gradually changed in time in the trough but not at the crest (**Figure 7a,b**); these trends were similar to those of the interaction strength (**Figure 6**). The average proportion of distances in the secondary peak was two times higher in the trough than at the crest (**Figure 7c, d**). Even though only relatively few cases (up to 4%) were contained within the secondary peak, the observed trends suggest that a certain cell population increasingly aggregated in time most likely due to a higher retention time of cells in the trough than at the crest.

## Discussion

Our combined findings from empirical observations and modelling suggest that biotic or chemical interactions drive the first steps from single primary cells towards cluster formation in nascent stream biofilms. Voronoi tessellation was sensitive enough to identify deviations from uniform-random distribution — patterns that could not have been resolved by simple visual inspection.

Microbial cells gradually deviated from uniform-random Poisson-Voronoi in the trough but not at the crest, which suggests hydrodynamic controls on microbial behaviour. As suggested by the ADV data, turbulent mixing — a random phenomenon by nature — rather than diffusion dominated the transport of cells and NLP in our system. The random motion is characterised by the microscopic length scales, and both cells and NLP would move around and between eddies (from millimetres to centimetres) to randomly deposit. However, before cells and NLP can deposit onto to the slides, they must pass through the boundary layer where nearly laminar conditions essentially determine exchange processes. It is reasonable to assume that randomly distributed seeds in the water column preserve their random signature when passing the laminar boundary layer, especially when random walk prevails [48].

Once deposited, the distribution of primary cells would thus remain nearly random in the absence of biotic and chemical interactions [21]. Essentially, this is what we found at the crest in a predominantly unidirectional and relatively structured (i.e., less fractal) flow environment where primary cells did not increasingly deviate from their initial distribution pattern. Whereas in a more erratic flow environment with alternating recirculation and elevated shear stress in the trough, the distribution of primary cells increasingly deviated from uniform randomness over the 12-h colonisation. We suggest that this is indicative of hydrodynamic-induced clustering. We propose that high-intensity turbulent eddies may erode the boundary layer in the trough more frequently than at the crest, which may perturb cells locally to eventually induce cell clustering. This would in fact be supported by the findings that even single cells can experience small-scale changes in turbulence [12], [43], [44] and that cluster formation can be a response to adverse environmental conditions [21], [49], [50]. Experimenting with *Pseudomonas aeruginosa* in microfluidics devices, it was also shown that shear stress could enhance adhesion and increase residence time [12], which may also facilitate cell-cell interactions and clustering.

Cell motility, chemotaxis or cell-to-cell signalling may lead to cluster formation. For instance, Park and colleagues [17], [18] found cell attraction induced by chemoattractants, which, in closed systems can lead to quorum sensing. Recently, Harris and colleagues [51] found directed movement by *Shewanella* towards a redox active source due to electrokinesis. Furthermore, EPS footprints that often exceed the size of the microbial cells themselves [41], [42] can adsorb and concentrate nutrients and chemoattractants from the bulk liquid and hence attract neighbouring cells [17], [18]. Ultimately, such processes would shift the spatial distribution from randomness to clustering as observed in closed systems [17], [18]. We suggest that similar self-attraction and spatial positioning of cells on homogenous surfaces in an open system, as used in this study, may enable cell-to-cell signalling even at low cell densities [40].

This scenario is supported by recent theoretical considerations [40] and empirical studies [52]–[54] showing that cells, although few and distant to each other, can communicate depending on their spatial positioning. Knowledge on cell-to-cell signalling in natural environments is admittedly poor [55]. The few reported “calling distances” range from a few micrometers in open systems, such as oral biofilms [53], to 78 µm in closed systems, such as in the rhizosphere [54]. These values bracket our measured distances of nearest neighbours, implying that cells in our system were located within a space potentially allowing cell-to-cell signalling. In open systems, such as streams, cell-to-cell signalling would have to occur within the boundary layer where advective flow does not remove signals [56].

Rather than using synthetic microbeads as a conservative tracer, we relied on naturally occurring NLP to compare cell patterns against. The observed divergence of NLP from cells and their higher interaction strength in the trough may be attributable to preferential adhesion of NLP to the EPS footprint from cells. Random walk of adhering microorganisms may gradually increase the footprint and hence the size of the patches conditioned with EPS. Furthermore, as shown for *Escherichia coli*, rolling adhesion can increase surface colonization in flowing environments [57] eventually leading to larger footprints. We suggest that NLP preferentially adhere to these patches and that several NLP can attach to one such patch if large enough. While cells remain randomly distributed unless entrained in gradient sensing [21], NLP would continue clustering.

In this study, we employed VT rather than weighted-average and distance-based methods (e.g. [27]). These methods are particularly suitable for uniformly distributed data, but less for anisotropic distributions as in our study, for instance [47]. Finding the appropriate distance to identify neighbours is difficult and requires *a priori* knowledge of the dataset. By contrast, natural neighbour interpolation, such as by VT, is not affected by these issues [46], [47]. A possible disadvantage of VT is that it is not possible to exactly localise the seed within a given polygon; this could lead to a bias in anisotropic polygons with a large area. Nevertheless, area is a more integrative measure than distance *per se* and better captures seed behaviour [33], [46].

In summary, our results suggest that Voronoi tessellation captured the first steps of primary cells towards clustering. Our model suggests that, depending on the hydrodynamic microenvironment, chemical or biotic interactions may induce the observed clustering. Such an accelerated transition from single primary cells towards clusters may be particularly advantageous in natural environments, such as streams, where the initial lag phase can be prolonged because of often harsh conditions (e.g., low resources) [58]. Our findings now open the avenue for future research with defined mixed populations under well-controlled conditions to further test some of the proposed mechanisms.

## Supporting Information

Figure S1 **Construction of the cumulative probability function (CPF).** The black squares give the cumulative probabilities of the actually measured and scaled Voronoi polygons. The grey triangles give the starting and end points of linearly interpolated CPF segments. Segments (a) and (d) are given by the appropriate lower and upper incomplete Γ-function, respectively, having no discontinuity at the segments (b) and (c). [*x_n_,,x _xn+1_*] depicts the interval for numerical differentiation (see equation 2, main text).
(TIF)

Figure S2 **Cylinder-like model.** The shape of the interaction strength is essentially given by *α* and determines the distribution of attachment probabilities within the area of interaction. A cylindrical volume given by α = 0 or α≫10, both with a statistically almost equal attraction probability along the radius.
(TIF)

Figure S3 **Contour plots of representative cell and NLP samples at the trough and the crest.** The color spectrum represents the 10% best fitted RSS values, where dark blue colors indicate best model fits, decreasing fits from light blue over green to yellow and orange. Generally, *r*_H_ values where less specific for cells than for NLPs, but interaction strength could be determined for both, NLPs and cells.
(TIF)

Figure S4 **Abundance of attached cells and NLP.** On PLL-coated slides more cell and NLPs were attached than on non-coated slides. Cells and NLP attached on the crest and in the trough in similar densities on the glass slides. Given are mean±SD.
(TIF)

Table S1 **Comparative statistics of Voronoi tessellation (VT) curves** ***versus*** **the respective Poisson Voronoi (PV) function and versus the best model fit.**
(DOC)
