# Supplementary figures and images for: Voronoi Tessellation Captures Very Early Clustering of Single Primary Cells as Induced by Interactions in Nascent Biofilms
Source: PLoS One. 2011 Oct 18;6(10):e26368. doi: 10.1371/journal.pone.0026368 (PMC3196551; doi:10.1371/journal.pone.0026368)

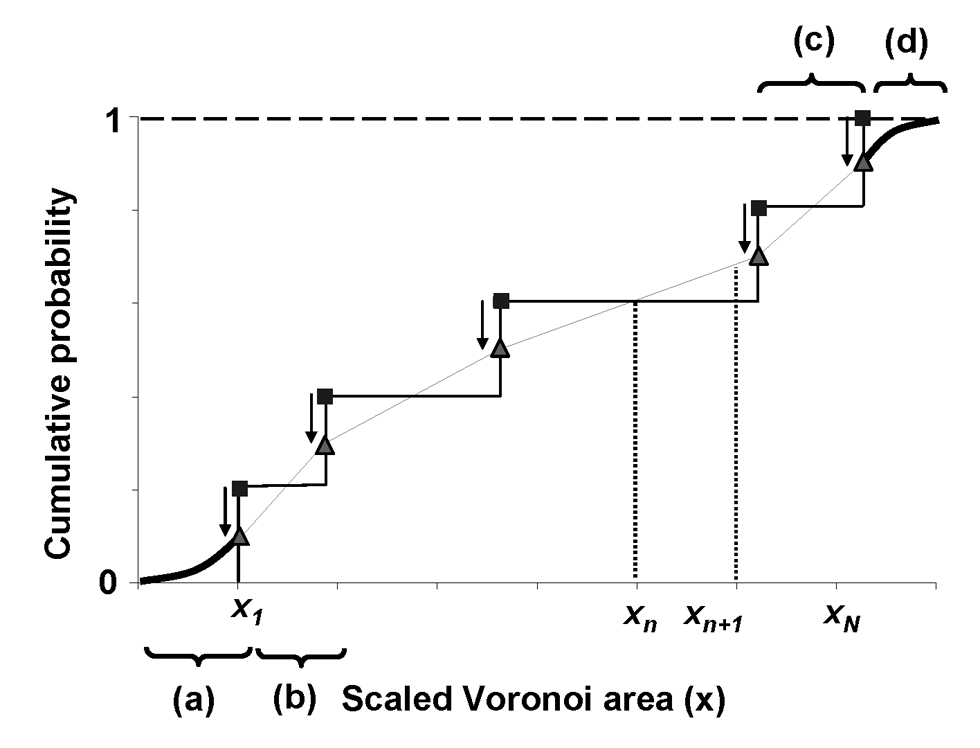

Supplement: Figure S1 — Construction of the cumulative probability function (CPF). The black squares give the cumulative probabilities of the actually measured and scaled Voronoi polygons. The grey triangles give the starting and end points of linearly interpolated CPF segments. Segments (a) and (d) are given by the appropriate lower and upper incomplete Γ-function, respectively, having no discontinuity at the segments (b) and (c). [xn,,x xn+1] depicts the interval for numerical differentiation (see equation 2, main text). (TIF) [file pone.0026368.s001.tif]

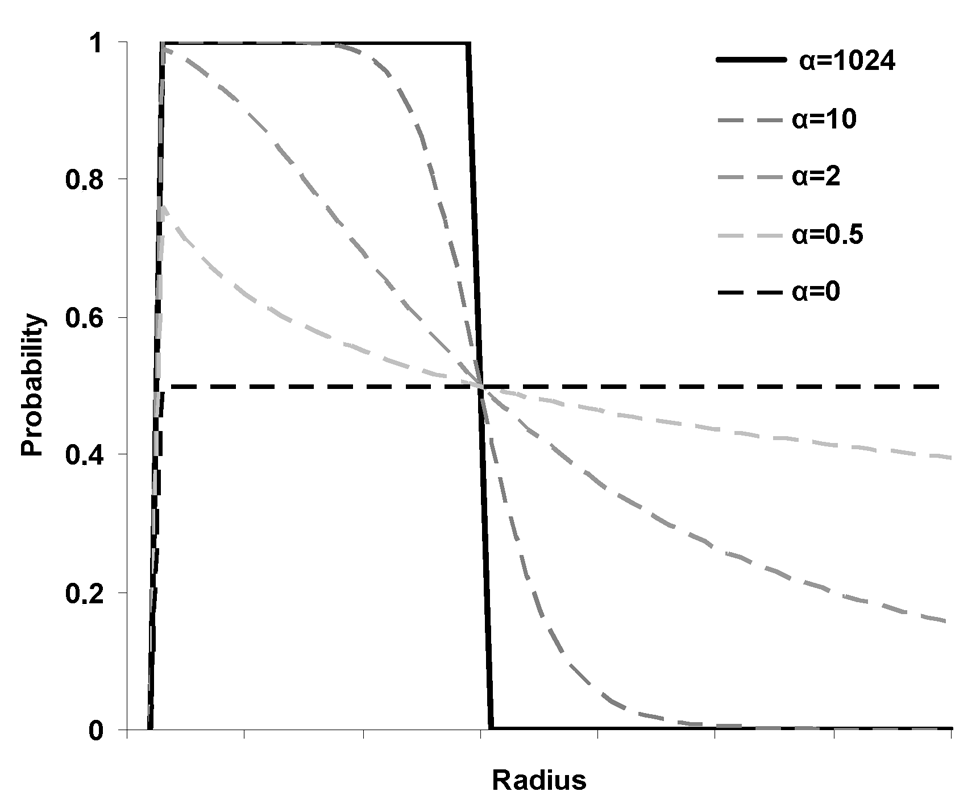

Supplement: Figure S2 — Cylinder-like model. The shape of the interaction strength is essentially given by α and determines the distribution of attachment probabilities within the area of interaction. A cylindrical volume given by α = 0 or α≫10, both with a statistically almost equal attraction probability along the radius. (TIF) [file pone.0026368.s002.tif]

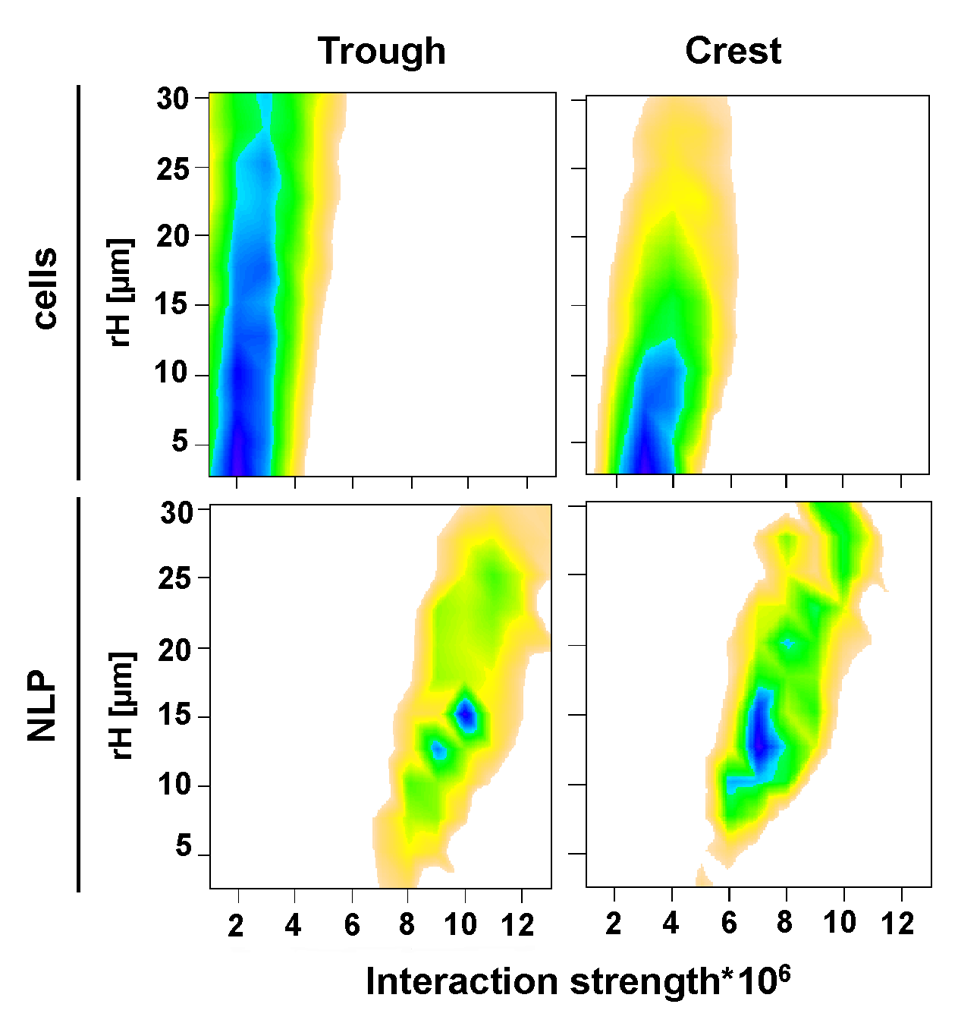

Supplement: Figure S3 — Contour plots of representative cell and NLP samples at the trough and the crest. The color spectrum represents the 10% best fitted RSS values, where dark blue colors indicate best model fits, decreasing fits from light blue over green to yellow and orange. Generally, r H values where less specific for cells than for NLPs, but interaction strength could be determined for both, NLPs and cells. (TIF) [file pone.0026368.s003.tif]

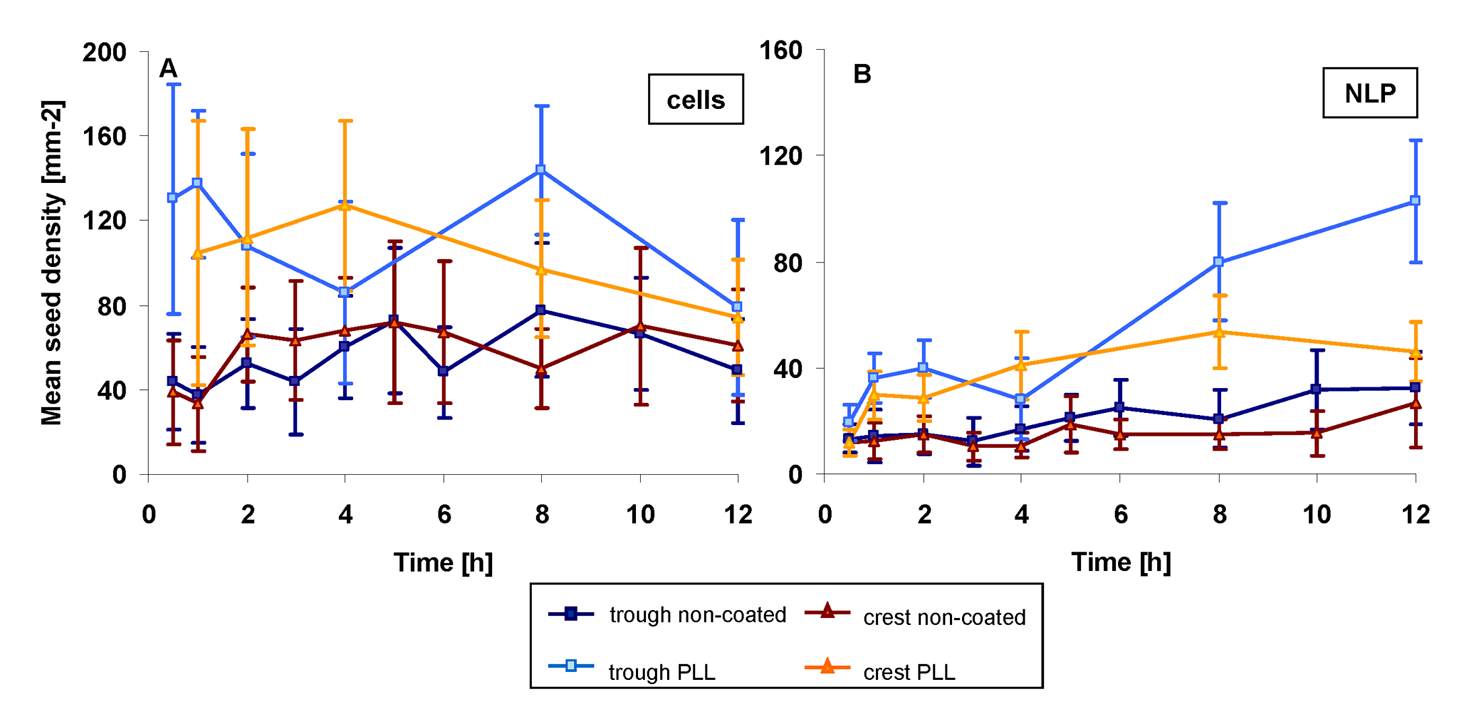

Supplement: Figure S4 — Abundance of attached cells and NLP. On PLL-coated slides more cell and NLPs were attached than on non-coated slides. Cells and NLP attached on the crest and in the trough in similar densities on the glass slides. Given are mean±SD. (TIF) [file pone.0026368.s004.tif]
