# Supplementary material for: Voronoi Tessellation Captures Very Early Clustering of Single Primary Cells as Induced by Interactions in Nascent Biofilms
Source: PLoS One. 2011 Oct 18;6(10):e26368. doi: 10.1371/journal.pone.0026368 (PMC3196551; doi:10.1371/journal.pone.0026368)
Supplement: Table S1 — Comparative statistics of Voronoi tessellation (VT) curves versus the respective Poisson Voronoi (PV) function and versus the best model fit. (DOC) [file pone.0026368.s005.doc]

**Table S1.**

| **seed** | **habitat** | **[h]** | **images** | **PV KS** | **p** | **V*10^6^** | **model RSS** |
| --- | --- | --- | --- | --- | --- | --- | --- |
| cell | crest | 0.5 | 81 | 0.0042 | <0.001 | 2.25 | 0.0362 |
| cell | crest | 1 | 94 | 0.0052 | <0.001 | 3 | 0.0299 |
| cell | crest | 2 | 115 | 0.0050 | <0.001 | 1 | 0.0241 |
| cell | crest | 3 | 69 | 0.0054 | <0.001 | 1.25 | 0.0317 |
| cell | crest | 4 | 81 | 0.0042 | <0.001 | 0.8 | 0.0284 |
| cell | crest | 5 | 98 | 0.0042 | <0.001 | 1 | 0.0283 |
| cell | crest | 6 | 75 | 0.0059 | <0.01 | 1.75 | 0.0328 |
| cell | crest | 8 | 75 | 0.0042 | <0.001 | 3 | 0.0344 |
| cell | crest | 10 | 50 | 0.0048 | <0.001 | 1.25 | 0.035 |
| cell | crest | 12 | 77 | 0.0484 | <0.001 | 1 | 0.0331 |
| cell | trough | 0.5 | 93 | 0.0378 | <0.001 | 1 | 0.0345 |
| cell | trough | 1 | 95 | 0.0417 | <0.001 | 1.5 | 0.0332 |
| cell | trough | 2 | 84 | 0.0542 | <0.001 | 2 | 0.0317 |
| cell | trough | 3 | 100 | 0.0112 | 0.34 | 3.75 | 0.0369 |
| cell | trough | 4 | 77 | 0.0576 | <0.001 | 2.25 | 0.0306 |
| cell | trough | 5 | 89 | 0.0093 | 0.35 | 2.25 | 0.0339 |
| cell | trough | 6 | 89 | 0.0071 | <0.05 | 3.25 | 0.0300 |
| cell | trough | 8 | 86 | 0.0542 | <0.001 | 1.25 | 0.0300 |
| cell | trough | 10 | 86 | 0.0081 | 0.14 | 3.25 | 0.0343 |
| cell | trough | 12 | 74 | 0.0909 | <0.001 | 4.25 | 0.0390 |
| NLP | crest | 0.5 | 81 | 0.0284 | 0.42 | 1.25 | 0.0418 |
| NLP | crest | 1 | 92 | 0.0433 | <0.05 | 5 | 0.0428 |
| NLP | crest | 2 | 113 | 0.0691 | <0.001 | 9.2 | 0.0362 |
| NLP | crest | 3 | 75 | 0.0406 | 0.15 | 6.25 | 0.0159 |
| NLP | crest | 4 | 80 | 0.0659 | <0.001 | 11.25 | 0.0366 |
| NLP | crest | 5 | 110 | 0.0785 | <0.001 | 10.75 | 0.0327 |
| NLP | crest | 6 | 78 | 0.0617 | <0.001 | 6.75 | 0.0720 |
| NLP | crest | 8 | 72 | 0.0553 | <0.01 | 5.75 | 0.0419 |
| NLP | crest | 10 | 71 | 0.0493 | <0.01 | 7 | 0.0496 |
| NLP | crest | 12 | 75 | 0.0827 | <0.001 | 6.75 | 0.0406 |
| NLP | trough | 0.5 | 92 | 0.0561 | <0.001 | 6.75 | 0.0317 |
| NLP | trough | 1 | 93 | 0.0549 | <0.001 | 5.5 | 0.0482 |
| NLP | trough | 2 | 79 | 0.0667 | <0.001 | 10.5 | 0.0400 |
| NLP | trough | 3 | 98 | 0.0618 | <0.001 | 14.5 | 0.0442 |
| NLP | trough | 4 | 73 | 0.0837 | <0.001 | 12 | 0.0371 |
| NLP | trough | 5 | 88 | 0.0855 | <0.001 | 10.5 | 0.0362 |
| NLP | trough | 6 | 82 | 0.0938 | <0.001 | 10 | 0.0351 |
| NLP | trough | 8 | 87 | 0.0967 | <0.001 | 10.5 | 0.0411 |
| NLP | trough | 10 | 86 | 0.1315 | <0.001 | 12.5 | 0.0331 |
| NLP | trough | 12 | 79 | 0.1376 | <0.001 | 14 | 0.0360 |
